# FLVCR1-related diseases: from clinical heterogeneity to mechanistic insights

**DOI:** 10.1093/braincomms/fcag165

**Published:** 2026-05-07

**Authors:** Diletta Isabella Zanin Venturini, Deborah Chiabrando

**Affiliations:** Department of Molecular Biotechnology and Health Sciences, Molecular Biotechnology Center (MBC) ‘Guido Tarone’, University of Torino, 10126 Torino, Italy; Department of Molecular Biotechnology and Health Sciences, Molecular Biotechnology Center (MBC) ‘Guido Tarone’, University of Torino, 10126 Torino, Italy

**Keywords:** pain, neuropathy, ataxia, retinitis pigmentosa, microcephaly

## Abstract

Feline Leukemia Virus subgroup-C Receptor 1 (FLVCR1) is an ubiquitously expressed choline and ethanolamine importer that is involved in the control of multiple aspects of cell biology including the regulation of phospholipids metabolism, heme homeostasis, mitochondria-ER contact sites and cellular bioenergetics. Mutations in the *FLVCR1* gene cause a spectrum of autosomal-recessive disorders mainly affecting the nervous system. Research conducted in the last decade highlighted the complexity of the clinical features associated with *FLVCR1* mutations, ranging from dysfunction of specific sensory modalities to severe neurodevelopmental defects. Despite important progress in understanding the FLVCR1 function, the molecular mechanisms responsible for the disease are still poorly understood and specific treatment for the affected patients is lacking. This review aims to critically examine the current knowledge surrounding FLVCR1-related diseases, from clinical manifestations to the underlying molecular mechanisms. We also propose future directions to advance research and improve patient treatment.

## Teaser

FLVCR1 is an ubiquitously expressed choline/ethanolamine transporter that regulates heme homeostasis, the contact sites between mitochondria and endoplasmic reticulum, and cellular bioenergetics. Autosomal-recessive mutations in *FLVCR*1 underlie a heterogeneous spectrum of disorders affecting the nervous system. This review synthesizes clinical and molecular insights and highlights priorities to advance research.

## Graphical Abstract

**Graphical Abstract:**
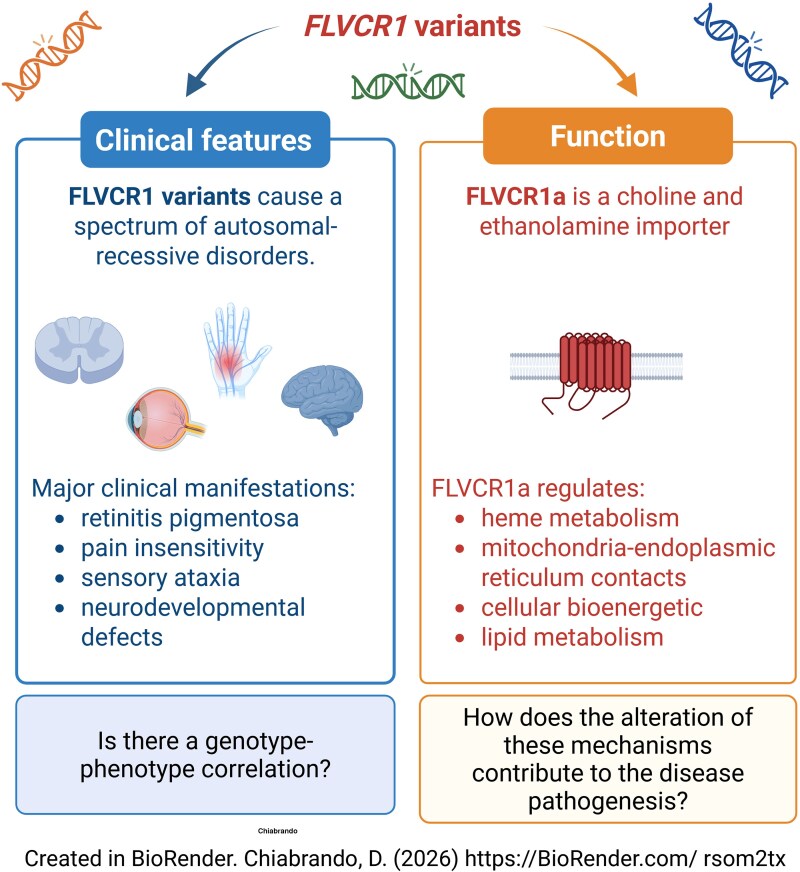

## Introduction

Mutations in the Feline Leukemia Virus subgroup-C Receptor 1 (*FLVCR1*) gene are responsible for a spectrum of rare autosomal recessive disorders that primarily affect the nervous system. Clinical manifestations range from mild dysfunction of sensory modalities (vision, proprioception and/or nociception) to severe neurodevelopmental defects, including microcephaly and hydrocephalus. Genetic and clinical heterogeneity have created inconsistencies in nomenclature, underscoring the need for a harmonized framework. Furthermore, the molecular mechanisms responsible for the disease are still poorly understood. While initial investigations described FLVCR1 as a heme exporter, recent studies have redefined FLVCR1 as an importer of choline and ethanolamine, with key functions in phospholipid metabolism, heme homeostasis, mitochondria–ER communication and cellular energy balance. Motivated by the convergence of expanding clinical reports and new mechanistic insights, we undertook a systematic review of the literature to provide a comprehensive overview of FLVCR1-related diseases. We summarize clinical features, genetic findings, and analyze genotype–phenotype correlations. Furthermore, we critically discuss potential pathogenic mechanisms and outline priorities for future research that will be essential to guide the development of targeted interventions.

## Flvcr1-Related Diseases

### Clinical features

Mutations in the *FLVCR1* gene were first identified in individuals affected by POSTERIOR COLUMN ATAXIA AND RETINITIS PIGMENTOSA (PCARP).^1^ PCARP has been defined as a childhood onset, autosomal-recessive neurodegenerative disorder,^2–4^ with a prevalence of less than 1 in 1 000 000 according to Orphanet. The disease manifests with night blindness (nyctalopia), reflecting a primary rod photoreceptor degeneration, followed by a gradual narrowing of the visual fields and a progressive decline in central retinal function over time, due to a secondary cone dysfunction. Peripheral visual field loss typically becomes apparent in late childhood or adolescence. Furthermore, the degeneration of the posterior columns of the spinal cord results in loss of proprioceptive sensation leading to ataxia that becomes clinically apparent in the second decade of life and progressively advances over time. In addition to retinitis pigmentosa and sensory ataxia, variable characteristics of the disease include scoliosis, camptodactyly, achalasia, gastrointestinal dysmotility, cataracts and mild sensory peripheral neuropathy.^1,2^ Since the initial description, there has been a continuous rise in the number of *FLVCR1* variants linked to PCARP.

While initial reports described sensory neuropathy as a variable feature of the disease, subsequent studies highlight pain-insensitivity as the major clinical finding in other patients. In these individuals, loss of pain perception occurs extremely early in infancy leading to the diagnosis of HEREDITARY SENSORY AND AUTONOMIC NEUROPATHY (HSAN).^5,6^ In these patients, the progressive degeneration of sensory neurons causes loss of nociception resulting in inadvertent self-injuries, persistent ulcerations, and frequently complicating factors such as soft tissue infections and osteomyelitis, often requiring amputations. Patients harboring *FLVCR1* mutations associated to profound pain-insensitivity may later experience varying degrees of sensory ataxia and/or retinitis pigmentosa. Furthermore, additional studies suggested that tremors,^7^ learning disability and developmental delay^8^ may represent additional varying clinical features of the disease.

Of note, some individuals carrying *FLVCR1* mutations develop only retinitis pigmentosa without any evidence of sensory ataxia, pain-insensitivity or other previously mentioned symptoms. Therefore, these patients have been diagnosed with NON-SYNDROMIC RETINITIS PIGMENTOSA (ns-RP).^9–12^

Genetic variations in the *FLVCR1* gene have recently been linked to a broader and more severe spectrum of developmental disorders. Calame *et al*.^13^ expanded the phenotypic understanding of *FLVCR1*-related disease by analyzing 30 individuals from 23 unrelated families with biallelic *FLVCR1* variants. While 21 patients exhibited the classic features reported above, the remaining 19 presented with a novel, severe neurodevelopmental phenotype. This included profound developmental delay, progressive microcephaly and brain malformations ranging from mild white matter loss to severe cortical atrophy and simplified gyral patterns, sometimes resembling hydranencephaly. Common features also included hypotonia, motor neuropathy, epilepsy, cortical visual impairment, optic atrophy, and spasticity. A subset of patients also showed intellectual disability and autistic features. Other individuals displayed self-injurious behaviour, sensory neuropathy or congenital insensitivity to pain, while retinitis pigmentosa and posterior column abnormalities were rare. The most severely affected cases had additional anomalies such as craniofacial and limb malformations, congenital heart and renal defects, and occasional macrocytic anemia.^13^ Consistently with these findings, we recently reported a fetus with profound microcephaly, hydrocephalus, and rudimentary cerebral structures, but no visceral anomalies.^14^

**Figure 1:**
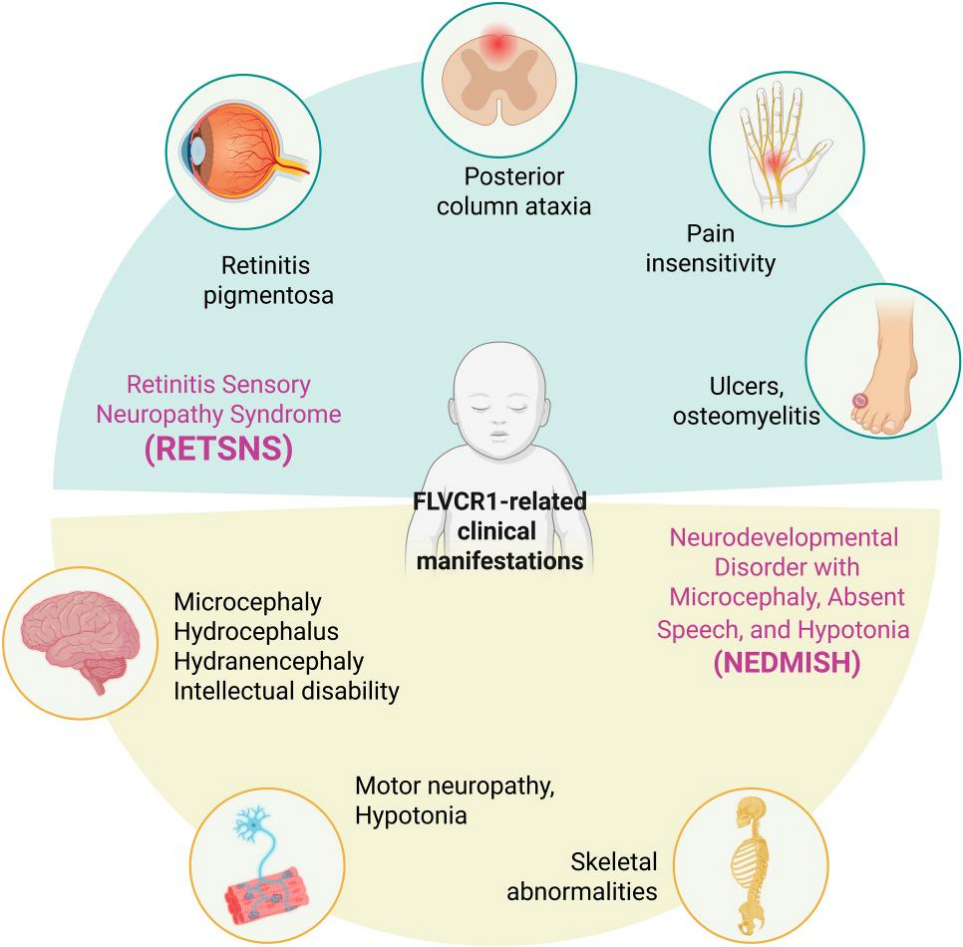
**Clinical features of FLVCR1-related diseases**. Clinical manifestations range from mild dysfunction of specific sensory modalities to more severe developmental defects. Common features include symptoms associated to sensory neuropathies like posterior column ataxia, retinitis pigmentosa and pain insensitivity. The progressive degeneration of sensory neurons that causes loss of nociception can result in inadvertent self-injuries like ulcers, mutilations and additional complicating factors like osteomyelitis. Furthermore, different mutations are correlated neurodevelopmental defects (microcephaly, hydrocephalus, hydranencephaly). Additional features include motor dysfunction, hypotonia and skeletal abnormalities. Created with BioRender. Chiabrando, D. (2026) https://BioRender.com/yyrx136.

Taken together, the data support two major clinical presentations associated with biallelic *FLVCR1* variants: a predominantly sensory/retinal phenotype, encompassing retinitis pigmentosa and/or sensory neuropathy with variable additional features, and a distinct, severe neurodevelopmental disorder (Fig. 1). This emerging framework has recently been reflected in the recently updated OMIM classification. Indeed, the term Retinopathy-Sensory Neuropathy Syndrome (RETSNS; #609033) was introduced to describe the individuals previously diagnosed with PCARP, HSAN or non-syndromic RP. This designation more accurately reflects the full spectrum of clinical features observed in these individuals and helps distinguish them from newly described patients with mutations in the same gene who present with a severe developmental disorder. This last category of patients was described with the new term Neurodevelopmental Disorder with Microcephaly, Absent Speech, and Hypotonia (NEDMISH; #621060). As the revised nomenclature has not yet been adopted in the published literature, we will, in the following sections, retain the terminology used in prior reports to facilitate direct comparison. Furthermore, we will additionally use the broader term FLVCR1-related diseases to encompass the full spectrum of clinical manifestations associated with *FLVCR1* mutations, and ‘FLVCR1-related neuropathy’ or similar terms to denote specific neurological phenotypes.

#### Molecular findings

FLVCR1-related diseases are rare autosomal recessive disorders. Advances in next-generation sequencing analyses have led to the identification of distinct types of *FLVCR1* variants. Missense variants occur in conserved transmembrane domains and are predicted to trigger conformational changes critical for protein function without affecting the plasma membrane localization of the protein.^6,13^ Frame-shift variants are predicted to result in the generation of truncated proteins that can be mis-localized or targeted for non-sense mediated decay (NMD), as demonstrated by *in vitro* studies on some overexpressed mutated proteins.^6,15^ Therefore, frame-shift variants might lead to loss of function alleles. Non-sense variants in the *FLVCR1* translation initiation codon (TIC) have been reported in three patients.^6,13^ TIC variants might escape NMD. TIC variants can affect protein translation in different manners leading to loss of translation initiation, *N*-terminal truncation or causing a shift in the mRNA reading frame.^16^  *In vitro* studies on the overexpressed mutated protein showed that the TIC variant in *FLVCR1* resulted in both reduced translation of the wild-type protein and translation initiation from a downstream ATG, leading to the production of an *N*-terminal truncated protein that is retained in the ER.^16^ These observations suggest that FLVCR1 function is reduced but not fully abrogated in patients carrying the biallelic TIC variants in *FLVCR1*. Finally, variants affecting the splicing of *FLVCR1* gene have been also reported. It is interesting to note the recurrence of the variant *FLVCR1* c.1092 + 5 A > G in patients diagnosed with ns-RP. The variant occurs in the intron between exons 4 and 5 of the *FLVCR1* gene, interferes with the correct splicing of *FLVCR1* leading to the skipping of exon 4 from the pre-mRNA. Exon 4 skipping is predicted to introduce a premature termination codon thus generating a truncated mRNA that is likely targeted for nonsense mediated decay.^9^ It has been proposed that some copies of correctly spliced mRNA could be retained which might be sufficient to prevent extraocular neurological symptoms.^9^ An additional splice variant has been reported in a patient showing RP together with other neurological manifestations.^13^ All the *FLVCR1* variants reported to date have been identified either in the homozygous state or in compound heterozygosity.

#### Genotype-phenotype correlation

The genetic and clinical heterogeneity observed in individuals with *FLVCR1* mutations led us to examine possible genotype–phenotype correlations. We reviewed the literature up to January 2026 and organized all the reported *FLVCR1* variants into five tables according to clinical presentation or diagnosis (Tables 1–5).

**Table 1:** *FLVCR1* variants associated with posterior column ataxia and retinitis Pigmentosa (PCARP)

FLVCR1 variant(s)	Zygosity	*N*	Type of mutation	Exon-Intron^a^	Clinical features^b^	Ref
c.361A > G (p.Asn121Asp)	Homozygous	16	Missense	Exon 1	Childhood-onset RP, posterior column ataxia, mild sensory neuropathy in some patients	^1,17,18^
c.721G > A (p.Ala241Thr)	Homozygous	4	Missense	Exon 1	Childhood-onset RP	^1^
					Posterior column ataxia	
c.574T > C (p.Cys192Arg)	Homozygous	2	Missense	Exon 1	Childhood-onset RP	^1^
					Posterior column ataxia	
c.1477G > C (Gly493Arg)	Homozygous	2	Missense	Exon 8	Childhood-onset RP,	^19^
					Posterior column ataxia.	
c.1547G > A (p.Arg516Gln) and c.1593 + 5 + 8delGTAA	Compound Heterozygous	3	Missense	Exon 9	RP	^20^
			Splice-site variant	Intron 9	Posterior Colum Ataxia	
					Sensory polyneuropathy**	
					severe impairment of sensation to all modalities, osteomyelitis, amputations, chronic pain (?)	
c.596T > C (p.Leu199Pro)	Homozygous	1	Missense	Exon 1	Childhood-onset RP, posterior column ataxia.	^21^
					Developmental delay and mild intellectual disability	
c.648C > A, p.(Phe216Leu) and c.733A > T, p.(Asn245Tyr)	Compound Heterozygous	1	Missense	Exon 1	Typical features of PCARP with later onset of RP (20 years)	^22^
			Missense	Exon 1		
c.369T > G, p.Phe123Leu and c.733A > G, p.Asn245Asp	Compound Heterozygous	1	Missense	Exon 1	Retinitis pigmentosa and sensory ataxia	^23^
			Missense	Exon 1		
	Total *N*=	30				

^a^ *FLVCR1* variants occurring in exon 1 are expected to affect exclusively FLVCR1a function, whereas variants occurring in the other exons/intron impact on both FLVCR1a and FLVCR1b isoforms.

^b^In this subset of patients, sensory neuropathy has been described as a variable feature of the disease. We advise readers to consult the original papers for a comprehensive description of each patient's clinical features.

*N* = number of affected individuals.

**Table 2:** *FLVCR1* variants associated with hereditary sensory and autonomic neuropathy (HSAN)

*FLVCR1* variants	Zygosity	*N*	Type of mutation	Exon-Intron^a^	Clinical features^b^	Ref
c.574T *>* C; p.(Cys192Arg) and c.610del; p.(Met204Cysfs*56)	Compound Heterozygous	1	Missense	Exon 1	HSAN	^5^
			Frame-shift	Exon 1		
c.661C *>* T; p.(Pro221Ser) and c.1324dup; p.(Tyr442Leufs*7)	Compound Heterozygous	1	Missense	Exon 1	HSAN	^5^
			Frame-shift	Exon 7		
c.661C > T, p.Pro221Ser	Homozygous	1	Missense	Exon 1	HSAN and PCARP	^24^
c.2T > C; p.(Met1Thr) and c.3G > T; p.(Met1Ile)	Compound Heterozygous	2	Start loss	Exon 1	HSAN	^6^
			Start loss	Exon 1		
c.3G > T; p.(Met1?) and c.730G > A; p.(Gly244Ser)	Compound Heterozygous	1	Start loss	Exon 1	HSAN and PCARP	^25^
			Missense	Exon 1		
c.371A > T; p.(Gln124Leu) and c.655G > T; p.(Gly219Cys)	Compound Heterozygous	1	Missense	Exon 1	HSAN	^26^
			Missense	Exon 1		
c.139_151del, p.(Phe47Glyfs*62) and c.722C > T, p.(Ala241Val)	Compound Heterozygous	1	Deletion/frame-shift	Exon 1	HSAN	^27^
			missense	Exon 1		
c.868_871del, p.(Ile290*) and c.655G > A, p.(Gly219Ser)	Compound Heterozygous	1	Deletion	Exon 2	HSAN	^27^
			Missense	Exon 1		
c.1318_1321del, p.(Thr440Valfs*63) and c.1317G > A, p.(Met439Ile)	Compound Heterozygous	1	Deletion/frameshift	Exon 7	HSAN	^27^
			Missense	Exon 7		
c.1194C > A, p.(Tyr398*) and c.1526–3C > T, p.?	Compound Heterozygous	1		Exon 5	HSAN	^27^
				Exon 9		
c.758T > A, p.(Phe253Tyr) and c.1369G > A	Compound Heterozygous	1	Missense	Exon 2	HSAN	^27^
			Missense	Exon 7		
c.1034C > G, p.(Thr345Ser)	Homozygous	1	Missense	Exon 4	HSAN	^27^
c.1022A > G (p.Tyr341Cys) and c.1307 + 5G > T	Compound Heterozygous	1	Missense	Exon 3	retinitis pigmentosa, sensory ataxia, scoliosis learning disability and developmental delay	^8^
			Splice-site variant	Intron 6		
c.498 G > A; p.(Trp166*) and c.369 T > G; p.(Phe123Leu).	Compound Heterozygous	1	Frameshift	Exon 1	sensory neuropathy, retinitis pigmentosa and tremors	^7^
			Missense	Exon 1		
c.1028T > C, p.(I343T)	Homozygous	2	Missense	Exon 4	sensory neuropathy	^13^
	Total *N* =	17				

^a^ *FLVCR1* variants occurring in exon 1 are expected to affect exclusively FLVCR1a function, whereas variants occurring in the other exons/intron might impact on both FLVCR1a and FLVCR1b isoforms.

^b^This subset of patients shows severe/early onset sensory neuropathy and therefore diagnosed with HSAN. They can manifest also typical symptoms of PCARP or additional features. We advise readers to consult the original papers for a comprehensive description of each patient's clinical features.

*N* = number of affected individuals.

**Table 3:** FLVCR1 variants associated with non-syndromic retinitis Pigmentosa (ns-RP)

*FLVCR1* variants	Zygosity	*N*	Type of mutation	Exon-Intron^a^	Clinical features^b^	Ref
c.479T > C; p.(Leu160Pro) and c.1092 + 5G > A	Compound Heterozygous	1	Missense	Exon 1	ns-RP	^9^
			Splice variant	Intron 4		
c.1092 + 5G > A	Homozygous	1	Splice variant	Intron 4	ns-RP	^9,10^
c.1285 T > C p.Phe429Leu in cis with c.1092 + 5G > A ^VUS^ and c.1092 + 5G > A	Homozygous	1	Splice variant	Exon 6	ns-RP	^10^
				Intron 4–5		
				Intron 4–5		
c.755del;p.(Gly252Alafs*8) and c.1092 + 5G > A	Compound Heterozygous	2	Deletion/Frameshift/Loss of function	Exon 2	ns-RP	^11^
			Splice variant	Intron 4		
c.847G > C;p.(Ala283Pro)^VUS^ and c.1092 + 5G > A	Compound Heterozygous	1	Missense	Exon 2	ns-RP	^11^
			Splice variant	Intron 4		
c.202C > T;p.(Gln68*) and c.1158T > G;p.(Ile386Met)^VUS^	Compound Heterozygous	1	Nonsense/Loss of function	Exon 1	ns-RP	^11^
			Missense	Exon 5		
c.1092 + 5G > A	Homozygous	1	Splice variant	Intron 4	ns-RP	^11^
c.1022A > G, p.Tyr341Cys	Homozygous	1	Missense	Exon 3	ns-RP	^12^
c.1022A > G, p.Tyr341Cys	Homozygous	7	Missense	Exon 3	ns-RP	^28^
	Total *N* =	16				

^a^ *FLVCR1* variants occurring in exon 1 are expected to affect exclusively FLVCR1a function, whereas variants occurring in the other exons/intron might impact on both FLVCR1a and FLVCR1b isoforms.

^b^ *FLVCR1* mutations may also cause isolated RP without signs of posterior column ataxia or pain-insensitivity (ns-RP). Note the recurrency of the splice-site variant c.1092 + 5G > A. We advise readers to consult the original papers for a comprehensive description of each patient's clinical features.

*N* = number of affected individuals.

**Table 4:** *FLVCR1* variants associated with milder neurodevelopmental defects

*FLVCR1* variants	Zygosity	*N*	Type of mutation	Exon-Intron^a^	Clinical features^b^	Ref
c.1_18del, p.(M1_D6del)	Homozygous	1	Start loss	Exon 1	mild intellectual disability, retinitis pigmentosa, motor and sensory n^e^uropathy, self-mutilation, osteomyelitis, hypotonia	^13^
c.382T > A, p.(Tyr128Asn)	Homozygous	1	Missense	Exon 1	retinitis pigmentosa, motor and sensory neuropathy	^13^
c.382T > A p.(Tyr128Asn)	Homozygous	1	Missense	Exon 1	progressive spastic paraplegia, motor neuropathy, scoliosis, hypotonia, normal IQ	^13^
c.757T > G, p.(Phe253Val)	Homozygous	2	Missense	Exon 2	severe developmental delay, intellectual disability, autistic features, short stature, microcephaly, hypotonia, hyporeflexia, normal EMG/NCV.	^13^
c.502C > G, p.(Leu168Val)	Homozygous	1	Missense	Exon 1	gross motor delay, normal cognition, impaired saccadic pursuit, sensory neuropathy	^13^
c.574T > C, p.(Cys192Arg)	Homozygous	1	Missense	Exon 1	mild developmental delay, sensory neuropathy, congenital insensitivity to pain, hypotonia, self-injury, and dysphagia	^13^
c.746C > G p.(Thr249Ser)	Homozygous	1	Missense	Exon 2	hypotonia and motor-sensory neuropathy	^13^
c.1028T > C p.(Ile343Thr)	Homozygous	1	Missense	Exon 4	pigmented retinopathy and dystonic head tremor	^13^
c.160dup p.(Arg54ProfsTer36)	Homozygous	2	Frameshift	Exon 1	Severe global developmental delay, hypotonia, pain insensitivity with widespread ulcers/scars.	^29^
					Neurotrophic keratopathy (bilateral corneal leukoma/clouding and absent corneal reflexes)	
c.687_688de (p. Phe229LeufsTer37)	Homozygous	1	Frameshift	Exon 2	type 1 diabetes mellitus, reduced visual acuity but not typical signs of RP, no sensory impairment, spasticity, peripheral axonal and motor neuropahy,	^30^
c.704C > A (p.Ser235Tyr) and c.1369G > A (p.Glu457Lys)	Compound Heterozygous	1	Missense	Exon 5	Pain insensitivity and self-injury complications, autonomic criss, delayed motor development, gait disturbance	^31^
			Missense	Exon 7		
c.1193A > G (p.Tyr398Cys)) and pseudogene insertion in intron 8	Compound Heterozygous	1	Missense	Exon 5	Early onset pain insensitivity, motor delay and visual disturbance	^31^
			Insertion	Intron 8		
Total *n* =		14				

^a^ *FLVCR1* variants occurring in exon 1 are expected to affect exclusively FLVCR1a function, whereas variants occurring in the other exons/introns might impact on both FLVCR1a and FLVCR1b isoforms.

^b^The table reported the variants associated with milder neurodevelopmental defects, according to Calame D.G. *et al*.^13^ We advise readers to consult the original paper for a comprehensive description of each patient's clinical features.

*N* = number of affected individuals.

**Table 5:** *FLVCR1* variants associated with severe neurodevelopmental defects

*FLVCR1* variants	Zygosity	*N*	Type of mutation	Exon-Intron^a^	Clinical features^b^	Ref
c.160delC, p. Arg54GlyfsTer59	Homozygous	1	frameshift	Exon 1	congenital hydrocephalus	^14^
c.1390G > A, p.(Gly464Ser)	Homozygous	1	missense	Exon 7	microcephaly and other neurodevelopmental defects, sensory neuropathy, visual impairment	^13^
c.1328T > G, p.(Leu443Pro)	Homozygous	1	missense	Exon 7	microcephaly and other neurodevelopmental defects	^13^
c.1169T > G, p.(Leu390*) and c.1261G > A, p.(Asp421Asn)	Compound Heterozygous	1	Loss-of-function (NMD)	Exon 5	microcephaly and other neurodevelopmental defects, retinopathy, scoliosis	^13^
			Missense	Exon 6		
c.153_154insC, p.(A52Rfs*38)	Homozygous	1	Loss-of-function (NMD)	Exon 1	microcephaly and other neurodevelopmental defects, retinitis pigmentosa	^13^
c.915T > G, p.(Ser305Arg)	Homozygous	1	Missense	Exon3	microcephaly and other neurodevelopmental defects, visual impairment	^13^
c.1235G > C, p.(Gly412Ala)	Homozygous	2	Missense	Exon 6	microcephaly and other neurodevelopmental defects. Older deceased brother was similarly affected.	^13^
c.1390G > A, p.(Gly464Ser)	Homozygous	1	Missense	Exon 7	microcephaly and other neurodevelopmental defects, hypotonia, retinitis pigmentosa, osteomyelitis, sensory neuropathy	^13^
c.1019C > T, p.(Thr340Ile) and c.1225T > C, p.(Ser409Pro)	Compound Heterozygous	3	Missense	Exon 3	microcephaly and other neurodevelopmental defects, hypotonia, scoliosis, macrocytic anemia	^13^
			Missense	Exon 6		
c.1593 + 5_1593 + 8del, p.(?)	Homozygous	3	Splice variant	Intron 9	microcephaly, ventriculomegaly and other developmental defects	^13^
c.218dup, p.(Glu74Arg fs*16)	Homozygous	1	Loss-of-function (NMD)	Exon 1	microcephaly, ventriculomegaly and other neurodevelopmental defects, hypotonia, sensorimotor polyneuropathy	^13^
c.1198C > T, p.(Gln400*) and c.884-3C > G, p.884_1024del, p.A295_Y341del	Compound Heterozygous	1	Loss-of-function (NMD)	Exon 6	microcephaly, cortical visual impairment, scoliosis, epilepsy, macrocytic anemia, bifid	^13^
			Splice-site (inframe deletion)	Intron 2		
c.1089T > G, p.(Tyr363*)	Homozygous	1	Loss-of-function (NMD)	Exon 4	Developmental defect	^13^
c.1235G > C, p. p.(Gly412Ala)	Homozygous	1	Missense	Exon 6	microcephaly, profound developmental delay, optic disk atrophy, hypotonia, and polydactyly	^13^
c.983T > C, p. p.(Leu328Pro)	Homozygous	1	Missense	Exon 3	Microcephaly and other neurodevelopmental defects, cortical visual impairment and hypotonia	^13^
c.1393_1402delCTTCTTAATGinsAC, p.(Leu465fs)	Homozygous	1	Frameshift	Exon 7	congenital hydrocephalus	^32^
Total *n* =		21				

^a^ *FLVCR1* variants occurring in exon 1 are expected to affect exclusively FLVCR1a function, whereas variants occurring in the other exons/introns might impact on both FLVCR1a and FLVCR1b isoforms.

^b^The table reported the variants associated with frofound developmental defects, according to Calame D.G. *et al*.^13^ We advise readers to consult the original paper for a comprehensive description of each patient's clinical features.

*N* = number of affected individuals.

**Figure 2:**
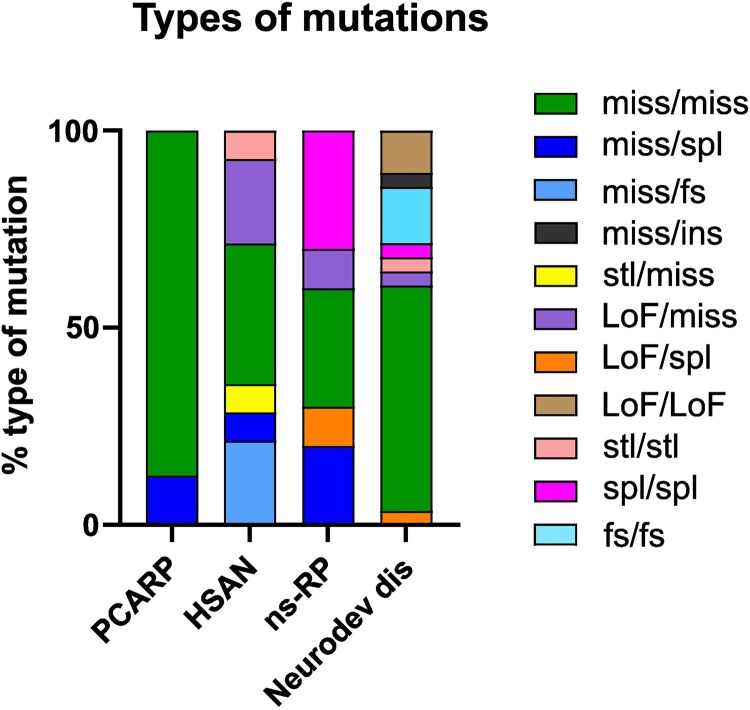
**Distribution of mutation types in posterior column ataxia and retinitis Pigmentosa (PCARP), hereditary sensory and autonomic neuropathy (HSAN), non-syndromic retinitis Pigmentosa (ns-RP), and neurodevelopmental disorders**. Bars represent the percentage contribution of each mutation class. Miss, missense; fs, frameshift; spl, splice variant; LoF, loss of function; stl, start loss; ins, insertion.

In patients with PCARP, ∼63% of variants occur in the homozygous state, with a clear predominance of missense mutations (Table 1 and Fig. 2). In contrast, among HSAN patients, about 80% of cases carry compound heterozygous variants, half of which consist of a frameshift mutation on one allele combined with a missense mutation on the other. Homozygous cases within this group exclusively involve missense variants (Table 2). In ns-RP, homozygous and compound heterozygous variants are observed at comparable frequencies; however, a recurrent splice variant affecting exon–intron 4 represents a characteristic feature (Table 3). Variants of *FLVCR1* associated with milder neurodevelopmental defects are found in more than 83% of cases in the homozygous state, predominantly missense mutations (Table 4). Curiously, homozygous missense *FLVCR1* variants are also predominant in individuals with severe neurodevelopmental defects with only four individuals harboring loss of function mutations in homozygosity (Table 5). In some circumstances the same mutation results in different clinical features in distinct individuals. For example the homozygous c.574T > C (p.Cys192Arg) variant have been identified in two patients showing pure PCARP^1^ and in another patient showing mild developmental delay, sensory neuropathy, congenital insensitivity to pain, hypotonia, self-injury and dysphagia.^13^

**Figure 3:**
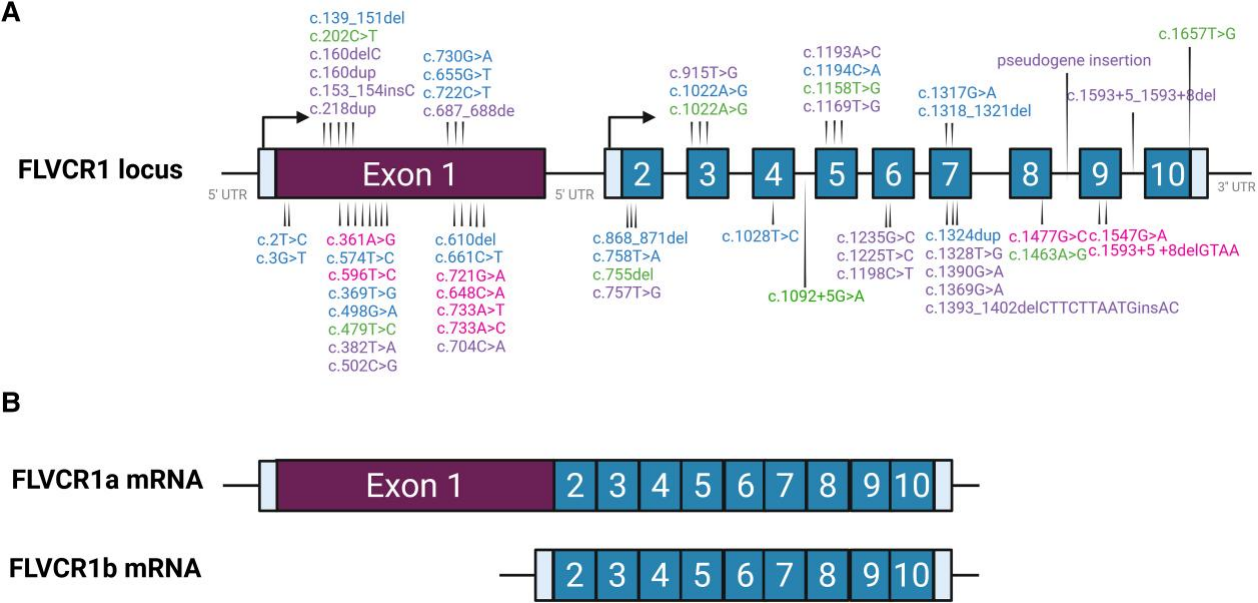
***FLVCR1* variants associated with FLVCR1-related diseases**. (**A**) *FLVCR1* gene locus and mutation localization. Different *FLVCR1* variants are reported on the respective exon or intron. Magenta: variants associated to Posterior Column Ataxia and Retinitis Pigmentosa (PCARP), Blue: variants associated to Hereditary Sensory and Autonomic Neuropathy (HSAN), Green: variants associated to non-syndromic Retinitis Pigmentosa (ns-RP), Violet: variants associated to neurodevelopmental disorders. (**B**) *FLVCR1a/b* mRNA. Created with BioRender. Chiabrando, D. (2026) https://BioRender.com/yyrx136.

The observation that homozygous missense mutations are linked to both the dysfunction of specific sensory modalities but also to neurodevelopment defects, suggests that the genomic location of the mutation might be associated with a different degree of FLVCR1 compromission. Alternatively, it has been proposed that environmental factors (poor maternal choline/ethanolamine intake) or genetic modifiers might be responsible for the different outcomes.^13^ Finally, phenotypic variations might depend on the impact of *FLVCR1* variants on different isoforms encoded by the *FLVCR1* gene. This gene encodes two distinct transcripts, likely arising from alternative transcription start sites. The full-length isoform, *FLVCR1a*, comprises 10 exons, whereas the shorter isoform, *FLVCR1b*, lacks exon 1 and includes all downstream exons.^33^ Pathogenic variants might affect FLVCR1a exclusively or impact both isoforms, depending on their genomic location. Therefore, we examined the exon distribution of *FVCR1* variants. Tables 1–5, Fig. 2 and Fig. 3 summarize all *FLVCR1* variants identified to date and their predicted effects on each isoform. We found that exon 1 (specific for FLVCR1a) is affected in ∼73% of PCARP cases and ∼56% of HSAN cases, regardless of zygosity, while the proportion decreases to ∼33% in neurodevelopmental defects and to ∼13% in ns-RP, the latter largely explained by the recurrence of the splice variant at exon–intron 4.

Overall, these findings suggest that zygosity, mutation type, and exon involvement may influence the clinical spectrum of FLVCR1-related disorders. Homozygous missense variants appear to underlie most PCARP cases, whereas compound heterozygous combinations, often missense plus frameshift, predominate in HSAN. A recurrent splice variant at exon–intron 4 characterizes ns-RP, while exon 1 emerges as a mutational hotspot in both PCARP and HSAN. Neurodevelopmental phenotypes are largely linked to homozygous missense variants, although occasional loss-of-function alleles are reported. These trends remain speculative and require confirmation in larger cohorts and functional studies.

##### Lessons from the study of animal models

Since its discovery, FLVCR1 has been recognized as essential for embryonic development, as constitutive loss of *Flvcr1* is incompatible with life. The first *Flvcr1* knockout mouse models that have been generated, presumably lacking both *Flvcr1a* and *Flvcr1b*, exhibited precocious embryonic lethality, skeletal malformation and impaired erythroid differentiation at the proerythroblast stage.^34^ The critical role of FLVCR1a in erythropoiesis was confirmed in conditional knockout mice lacking Flvcr1a postnatally in the bone marrow,^34,35^ as well as in zebrafish embryos upon *flvcr1* downmodulation.^35^ The analysis of *Flvcr1a*-specific knockout embryos also revealed early lethality and skeletal defects, and uncovered an additional role for FLVCR1a in angiogenesis,^33^ a role that was confirmed in endothelial-specific *Flvcr1a*-null embryos,^16,36^ as well as in zebrafish morphants.^16^ Although angiogenic defects have not been reported in FLVCR1-related disorders, skeletal malformations and macrocytic anemia have been described in few patients.^13^ Additional *in vivo* studies revealed that FLVCR1 function is required in adulthood for the proper function of several tissues including intestine,^37^ liver^38^ and muscle^39^ (Tables 6 and 7). However, aside from hypotonia and motor neuropathy described in a minority of patients, overt dysfunction of non-neural organs has not been a prominent feature of FLVCR1-related disease. Therefore, *Flvcr1* knockout embryos^33,34^ and *flvcr1a* morphants^35^ phenocopy some of the clinical features present in those patients with the most severe developmental defects (Tables 6 and 7). The embryonic lethality observed upon complete *Flvcr1* loss supports the notion that most disease-associated *FLVCR1* variants, particularly those linked to sensory neuropathy and/or retinitis pigmentosa, are likely hypomorphic rather than null. Residual FLVCR1a activity may be sufficient to sustain essential functions in non-neural tissues, and/or compensatory mechanisms in humans may mitigate the impact of partial FLVCR1 deficiency outside the nervous system.

**Table 6:** Mouse models generated to study the function of FLVCR1 during development and in adult tissues**4**This format of the table is clear whereas the tables I saw on the PDF proof are not clear because it is difficult to see where the description of one model finish and the other start. It is necessary to introduce a space. The same for the other tables on the PDF proof

Mouse models	Phenotype	Ref
Flvcr1 −/− embryos	Presumably Flvcr1a and Flvcr1b deletion Intrauterine deaths at or before embryonic day 7.5 (E7.5) and between E14.5 and E16.5. Abnormal limb, hand, and digit maturation; flattened faces; and hypertelorism. Normal cardiac, pulmonary, and genitourinary systems Impaired erythroid differentiation at the proerythroblast stage	^34^
Flvcr1a-null embryos	Intrauterine deaths around E14.5 Multifocal and extended hemorrhages, evident in the primordial limbs at E12.5 and then throughout the head and body, associated with subcutaneous edema Impaired angiogenesis Skeletal malformations	^33^
Postnatal deletion of Flvcr1 in the bone marrow	Severe hyperchromic macrocytic anemia and reticulocytopenia Cardiomegaly Splenomegaly	^34^
		^35^
Endothelial cells-specific Flvcr1a-null mice	Intrauterine deaths between E14.5 and E16.5 Intraembryonic bleeding, particularly the developing limbs, tail, and in the yolk sac Edema Skeletal malformations Defective developmental angiogenesis	^36^
		^16^
Inducible endothelial cells-specific Flvcr1a-null mice	Defective adult neo-angiogenesis (tumor angiogenesis)	^16^
Intestine-specific Flvcr1a-null mice	Decreased intestinal cell proliferation Impaired survival of mutant mice upon induction of ulcerative colitis	^37^
Hepatocyte-specific	Reduced expression and activity of cytochromes P450	^38^
Flvcr1a-null mice		
Skeletal-muscle-specific	Decreased heme synthesis in skeletal-muscle Deranged myofibre features and decreased overall muscle performance Alteration of muscle fibre metabolism, mitochondrial integrity and myoglobin expression Untimely satellite cell differentiation and compromised muscle regeneration	^39^
Flvcr1a-null mice		
Neural progenitors-specific Flvcr1a-null mice	Perinatal lethality Microcephaly, ventriculomegaly, and increased subarachnoid and perivascular spaces Reduced proliferation of neural progenitors	^14^
Retina-specific FLVCR1−/− mice	Early onset degeneration of the retina that manifest by P14 Reduced thickness of both outer nuclear layer (OLN) and inner nuclear layer (INL) Reduction of dark-adapted ERG responses Microglia infiltration and apoptosis Abnormal mitochondria structure	^40^
Rod-specific FLVCR1−/− mice	Early onset degeneration of the retina that begins around P25 ONL-specific thinning Photoreceptors degeneration with secondary cone loss at P30 Microglia infiltration and apoptosis Abnormal mitochondria structure	^40^

**Table 7:** Zebrafish models generated to investigate FLVCR1 function

Zebrafish models	Phenotype	Ref
flvcr1a/1b morphants	All morphants died within 5 days post-fertilization Developmental delay Shorter body, ventrally bent tail and smaller heads Hydrocephalus and lacked yolk extension Impaired erythropoiesis; anemia Defective intersegmental vessels formation	^35^
		^16^
*flvcr1a morphants*	Developmental delay Anemia Defective intersegmental vessels formation Reduced DRG number Reduced spontaneous movement Reduced swimming distance in response to tactile stimulation	^35^
		^16^
		^31^
*flvcr1 crispants*	Presumably targeting both *flvcr1a* and *flvcr1b* Reduced DRG number	^31^

Most individuals with FLVCR1-related disease exhibit clinical features primarily affecting the central or peripheral nervous system. Gross analyses of constitutive *Flvcr1* knockout embryos^33,34^ did not reveal overt abnormalities in early central nervous system patterning, suggesting that FLVCR1 may be dispensable for initial stages of brain development. In line with these observations, in situ hybridization showed the expression of *Flvcr1* in mouse neural tissues starting from E12,5^34^ and additional studies demonstrated that FLVCR1 is highly expressed at early stages of neocortical development during early neocortical development in both humans and mice before declining at later stages.^14^ Notably, conditional deletion of Flvcr1a in neural progenitors causes perinatal lethality with severe microcephaly and hydrocephalus, closely paralleling patients with the most severe neurodevelopmental presentations.^14^ Of note, hydrocephalus was also reported in zebrafish upon downmodulation of Flvcr1.^35^ Mechanistically, Flvcr1a is required to sustain neural progenitor proliferation during corticogenesis, and its loss promotes premature neuronal differentiation and cell death, likely contributing to cortical thinning and secondary ventricular enlargement.^14^ Therefore, the conditional deletion of *Flvcr1a* in neural progenitors provide a relevant model to investigate the mechanisms underlying neurodevelopmental defects (Tables 6 and 7).

Concerning FLVCR1-associated retinitis pigmentosa, studies in mouse models have shown that FLVCR1 is expressed predominantly in photoreceptors, with strong localization to the inner segments and additional signal in the outer plexiform layer.^40^ A recent study generated conditional knockout mouse models lacking *Flvcr1* either throughout the retina or specifically in rods. Retina-specific *Flvcr1* knockout mice exhibit early-onset, severe retinal degeneration characterized by progressive thinning of both the inner and outer nuclear layers and profound loss of ERG responses.^40^ In contrast, rod-specific Flvcr1 knockout mice develop retinal degeneration largely confined to rods and the outer nuclear layer, with reduced electroretinography responses but preserved inner nuclear layer thickness.^40^ Both mouse models display increased microglial activation and apoptosis during disease progression,^40^ (Tables 6 and 7) supporting their utility as relevant animal models to investigate the molecular mechanisms underlying retinitis pigmentosa.

In the context of FLVCR1-linked sensory neuropathy, animal models allowing sensory neuron–specific investigation of FLVCR1 function are currently lacking. Initial functional insights have instead been obtained from zebrafish, where downmodulation of *flvcr1a* results in a reduced number of dorsal root ganglia, a phenotype that is also recapitulated in *flvcr1* crispants.^31^ Consistent with these anatomical defects, *flvcr1a* morphants display marked functional impairments, including decreased spontaneous movements and reduced swimming distance in response to tactile stimulation, indicative of defective sensory perception (Tables 6 and 7).^31^ Collectively, these findings highlight the need for mammalian models enabling cell type–specific interrogation of FLVCR1 function in the peripheral nervous system.

## FLVCR1 Function and Pathogenetic Implications

### FLVCR1a structural insights and substrate specificity

FLVCR1a is a member of the major facilitator superfamily (MFS) of transporters. Early work described FLVCR1a as a 555-amino-acid protein with a molecular mass between 55 and 70 kDa. It has a 12-transmembrane domain structure with six hydrophilic extracellular loops and cytosolic *N*- and C-terminals.^41–43^

Building on these initial findings, the recent definition of FLVCR1a structure via cryo-electron microscopy (cyo-EM) has significantly deepened our understanding of FLVCR1a’s structure and function. A resolution of a 2.6 Å confirmed that FLVCR1a consists of two 6TM domains, flexibly connected by an intracellular linker.^44^ The first 6TM domain (TM1 to TM6) forms the amino-terminal domain, whereas the second domain (TM7 to TM12) forms the carboxy-terminal region. At the centre of FLVCR1a, a pseudo 2-fold symmetry axis is created by the homologous *N*- and C-terminal regions. Along this axis, a 30 Å central cavity, lined by electronegative residues, extends from the cytosolic side, suggesting a pathway suitable for substrate transport.^45^

Complementing these findings, the inward-facing conformation of FLVCR1a has been resolved at a 2.9 Å resolution.^46^ The analysis confirmed the presence of a wedge-shaped cavity ∼23 Å deep, created by separation between TM4 and TM5 in the *N*-terminal region and TM10 and TM11 in the C-terminal region. This structural feature strengthens the idea that FLVCR1a’s structure may be uniquely adapted for a specific substrate.

Heme and other cyclic planar porphyrins, such as protoporphyrin IX and coproporphyrin, has been initially identified as specific substrates exported by FLVCR1a.^44,47^ Interestingly, the heme export ability of FLVCR1a was found to be dependent on the avidity and concentration of extracellular heme-binding proteins, like hemopexin and albumin.^34^ Although no structural data of the heme–protein complex or real-time analyses of FLVCR1a-mediated heme transport are currently available, subsequent research has consistently demonstrated that FLVCR1a is undeniably implicated in the regulation of heme metabolism.^48^

On the other hand, many recent publications described FLVCR1a as a choline and ethanolamine transporter. The first evidence comes from structural understanding. Indeed, the above stated structural findings prompted further investigation into FLVCR1a’s role as a transporter of specific substrates. Son Y. and colleagues,^45^ building on their structural data, provided the first cryo-EM structure of FLVCR1a in the presence of choline, revealing the details of the choline-binding site. They found that the hydroxyl group of choline interacts with the side chains of Gln214 and Glu471, while its quaternary amine interacts with Trp125 and Tyr349. Additional residues lining the cavity, such as Tyr153, Met154 and Asn245, further stabilize choline binding. Interestingly, an almost identical substrate-binding site was observed even in the absence of exogenous choline, suggesting FLVCR1a’s high affinity for endogenous choline.

More specifically, choline occupies a central position in FLVCR1a’s cavity between the two 6TM domains where the Trp125 on TM1 interacts directly with choline, while aromatic residues Tyr349 and Phe348 contribute to stabilizing the binding.^46^ Interestingly, both choline and ethanolamine bind to this site, with ethanolamine occupying a deeper position in the aromatic pocket, specifically involving residues Trp125, Tyr153 and Tyr349. This distinction further elucidates how FLVCR1a might transport both molecules but with distinct binding orientations and interactions.^46^ Notably, the importance of Tyr349 residue in choline transport was previously anticipated by Alpha Fold prediction, still without FLVCR1a structure solution, using a peculiar organelle selective labelling screening approach.^49^

The structural insights were supported by functional assays in cell lines. In 2023, radiolabeled choline uptake assays in FLVCR1a knockout HEK293T and HeLa cells, showed that FLVCR1a knockout significantly impaired choline uptake in a dose and time-dependent manner.^50^ The phenotype could be rescued by expressing either FLVCR1a or the high-affinity choline transporter SLC5A7/CTH1. The same detrimental effect of FLVCR1a knockout in choline and ethanolamine transport was confirmed in further studies where the key residues identified in their cryo-EM structure (Trp125, Tyr153, Gln214 and Tyr349) for choline binding, into alanine to assess their role in regulating substrate transport through a radiolabeled choline/ethanolamine uptake assay.^45^ Trp125 emerged as essential residue for both metabolite transport, as well as Tyr153 and Tyr349 confirmed their critical role. On the other hand, Gln214 turned out to be critical for ethanolamine transport but not for choline uptake. Taken together, these studies demonstrated FLVCR1a as a transporter of choline and ethanolamine, although the residues specifically involved in regulating the transport vary depending on the substrate chemical properties.

#### FLVCR1a and phospholipids metabolism

Choline and ethanolamine are essential nutrients that play critical roles in cell structure and function. They are primarily involved in membrane phospholipid synthesis, but also in lipid metabolism, cell signaling, and in the maintenance of cellular homeostasis. Indeed, choline and ethanolamine are essential for cellular metabolism: they serve as precursors for phospholipid synthesis via the Kennedy pathway, and choline additionally contributes to acetylcholine production and to betaine formation, supporting neurotransmission methylation reactions and one-carbon metabolism.

**Figure 4:**
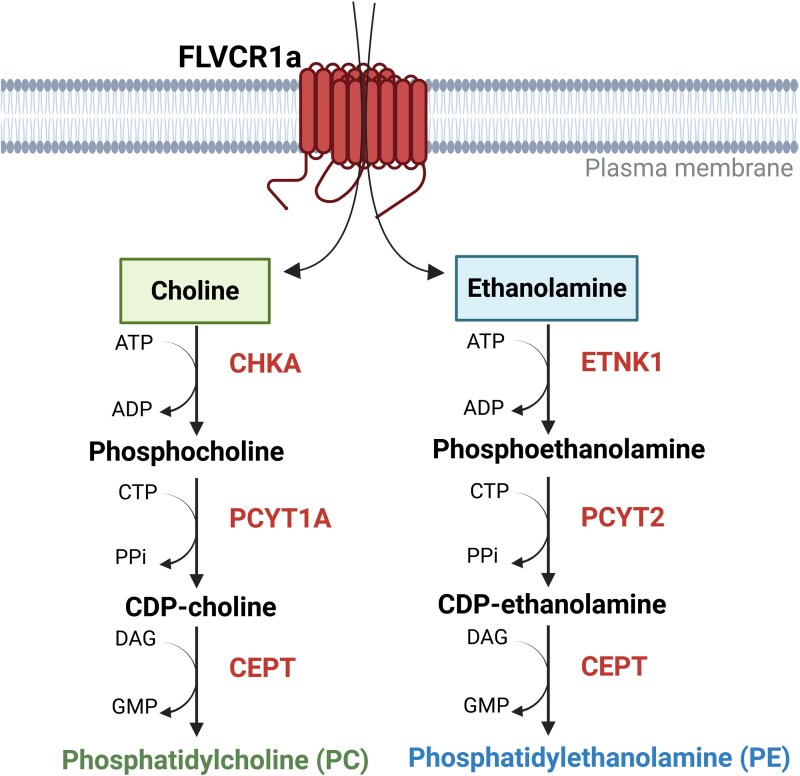
**Kennedy pathway**. FLVCR1a dependent choline and ethanolamine uptake contributes to the two branches of the Kennedy pathway. In the choline branch, choline is phosphorylated by choline kinase (CHKA) to form phosphocholine, which is then converted to cytidine disphosphate choline (CDP-choline) CTP:phosphocholine cytidylyltransferase (PCYT1A). Finally, CDP-choline is combined with diacylglycerol (DAG) by choline/ethanolamine phosphotransferase (CEPT) to produce Phosphatidylcholine (PC). Similarly, in the ethanolamine branch, ethanolamine undergoes phosphorylation by ethanolamine kinase to generate phosphoethanolamine, which is converted to CDP-ethanolamine by CTP:phosphoethanolamine cytidylyltransferase (PCYT2). CDP-ethanolamine is then used by CEPT to synthesize Phosphatidylethanolamine (PE). Created with BioRender. Chiabrando, D. (2026) https://BioRender.com/yyrx136.

Focusing on phospholipid metabolism, it is known that choline and ethanolamine are integral to the two branches of the Kennedy pathway (Fig. 4), a fundamental metabolic route for synthesizing the major phospholipids phosphatidylcholine (PC) and phosphatidylethanolamine (PE).^51^ In the choline branch, choline is phosphorylated by choline kinase (CHKA) to form phosphocholine, which is then converted to cytidine diphosphate-choline (CDP-choline) by CTP:phosphocholine cytidylyltransferase (PCYT1A). Finally, CDP-choline is combined with diacylglycerol (DAG) by choline/ethanolamine phosphotransferase (CEPT) to produce PC. Similarly, in the ethanolamine branch, ethanolamine undergoes phosphorylation by ethanolamine kinase to generate phosphoethanolamine, which is converted to CDP-ethanolamine by CTP:phosphoethanolamine cytidylyltransferase (PCYT2). CDP-ethanolamine is then used by CEPT to synthesize PE.

Consequently, FLVCR1a-mediated ethanolamine and choline transport could directly contribute to both branches of the Kennedy pathways. Indeed, metabolite isotope tracing experiments with isotopically labelled choline and ethanolamine in FLVCR1a-knockout cells, showed a marked reduction of choline incorporation into downstream metabolites like phosphocholine and betaine and similarly of ethanolamine into phosphoethanolamine, suggesting that FLVCR1a-mediated choline and ethanolamine transport is crucial for the Kennedy pathway.^45^ Similarly, a choline transport assay in A549 cell line silenced for FLVCR1a showed significantly reduced choline import and consequent reduced phosphatidylcholine and sphingomyelin levels compared with controls.^52^ Moreover, a LC-MS based study profiled the metabolism of FLVCR1a KO or complemented cell lines.^50^ FLVCR1a deficient cells showed significant reduction in choline, phosphocholine and betaine levels but also a strong accumulation of triglycerides and thus lipid droplets. However, FLVCR1a-knockout cells did not affect CHKA function and activity suggesting that FLVCR1a could primarily affect processes acting upstream of CHKA. A further key publication showed that loss of FLVCR1a in different cell lines is associated to a dramatic reduction of endogenous choline levels as well as of PC levels.^49^ In this study, authors developed a method to convert organelle specific lipid phenotypes into an accessible fluorescence signal for genome wide screening, called organelle selective click chemistry coupled with flow cytometry (O-click FC), to quantify the subcellular distribution of PC in the cell. This approach managed to select FLVCR1a as a gene involved in choline and PC metabolism.

Similarly, choline and ethanolamine metabolite profiles resulted affected in a comprehensive metabolomic analysis in FLVCR1a-knockout liver samples.^46^ Specifically, in FLVCR1a deficient samples the relative choline levels resulted higher compared with controls, whereas CDP-choline and phosphocholine levels lower. Moreover, CDP-ethanolamine levels were reduced whereas phosphoethanolamine levels were enhanced in knockout samples compared with controls ones.

Building on these new discoveries regarding the involvement of FLVCR1a as a transporter of choline and ethanolamine and its role in the Kennedy pathway, it would be valuable to investigate whether similar defects have been observed with FLVCR1a mutations associated with disease phenotypes to investigate whether the choline transport function of FLVCR1a is affected by these mutations.

Interestingly, 75% of disease associated variants overexpressed in FLVCR1a KO cells exhibited significantly reduced levels of PC, suggesting that disease associated mutations could perturb structural states thus leading to an impaired choline transport activity.^49^ Similarly, mutagenesis assays confirmed that different missense mutations resulted in impaired choline uptake.^52^ Notably, compared with the native protein, transport activity of mutated isoforms was in the range of 0–57% but this was not due to reduced expression levels. This evidence suggests that choline defect could contribute at least partially to the disease pathogenesis. Interestingly, the same trend was observed also in the newly identified FLVCR1a variants associated to sensory neuropathies as PCARP and HSAN studies.^13^ However, no significant difference in the transport activity was found between mild and severe phenotype associated variants.

In summary, it is important to note that, although the studies cited above have provided valuable insights into FLVCR1a and choline/ethanolamine transport, they have not employed physiological models of FLVCR1a-related disease, nor have they analyzed cells with endogenous FLVCR1a expression levels. However, preliminary evidence comes from studies conducted on patient-derived fibroblasts, which revealed reduced choline levels and altered membrane fluidity.^31^ Further studies will be important to confirm these observations and to assess whether targeting FLVCR1a activity could have therapeutic value in choline-related disorders. In addition, choline and ethanolamine are involved not only in the Kennedy pathway but also in acetylcholine synthesis, betaine pathways, one-carbon metabolism and membrane phospholipid production, although their precise roles in disease pathogenesis still need to be clarified experimentally.

#### FLVCR1a and heme metabolism

Based on the initial identification of FLVCR1 as a heme exporter, it has been proposed that the impairment of FLVCR1a function may cause heme accumulation and cellular damage by heme toxicity.^5,15,24^ However, experimental evidence in support of this hypothesis is poor. Studies on patients’ fibroblasts and lymphoblastoid cells failed to demonstrate heme accumulation at the steady state.^5,24^ Heme overload was detected in patients’ cells only following the stimulation of endogenous heme synthesis. While this observation was interpreted as indirect evidence of FLVCR1a’s role in heme export, we cannot rule out the possibility that the heme accumulation in patients’ cells upon stimulation of heme synthesis instead reflects an indirect consequence of altered membrane composition. Specific studies are required to address this issue. Despite discrepancy in the definition of the substrate specificity of FLVCR1a, it is undoubtedly evident that modulation of FLVCR1a affects heme metabolism.^48^ Indeed, studies showed that FLVCR1a function is essential to control the rate of endogenous heme biosynthesis.^53^ Although the underlying molecular mechanism require further investigation, it has been demonstrated that FLVCR1a controls the activity of the rate-limiting enzyme in heme biosynthesis ALAS1 (5-aminolevulinate synthase 1).^53^ Interestingly, reduced ALAS1 activity was reported also in patient-derived fibroblasts,^31^ suggesting that heme deficiency, rather than heme overload, may contribute to disease pathogenesis, with potential consequences for heme-dependent processes.

#### FLVCR1a at mitochondria associated membranes

**Figure 5:**
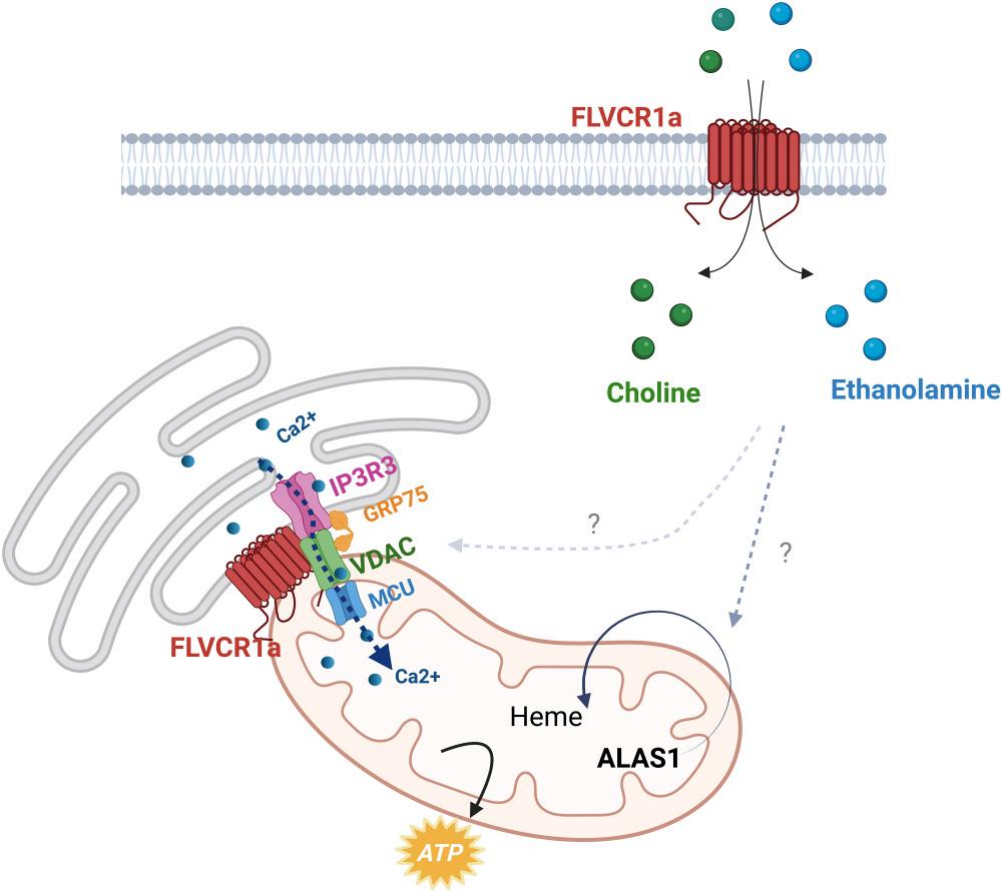
**Functions of FLVCR1a**. FLVCR1a has been described as a choline/ethanolamine importer with a crucial role in the regulation of intracellular heme metabolism. Modulation of FLVCR1a controls the activity of the rate-limiting enzyme in heme biosynthesis ALAS1 (5-aminolevulinate synthase 1), through a still elusive mechanism. More recently, evidence indicated that FLVCR1a interacts with the IP3R3–VDAC–GRP75 protein complex, located at MAMs and responsible for calcium transfer. Whether the roles of FLVCR1a as a choline/ethanolamine transporter and as IP3R3–VDAC–GRP75 protein complex are functionally interconnected or independent remains to be determined. Created with BioRender. Chiabrando, D. (2026) https://BioRender.com/yyrx136.

In parallel with the discovery of FLVCR1a's role as a choline and ethanolamine importer, new evidence from our recent research provides critical insights into the protein's function at mitochondria-associated membranes (MAMs)^14^ (Fig. 5). Remarkably, for the first time, FLVCR1a has been identified not only on the plasma membrane but also intracellularly, with multiple experimental approaches revealing its specific enrichment at ER-mitochondria contact sites. By analyzing the FLVCR1a interactome, using tandem affinity purification (TAP) coupled with mass spectrometry, we discovered that the top interactors of FLVCR1a are predominantly components of mitochondria, ER and MAMs. Among these, IP3R3, VDAC and GRP75—key constituents of the calcium transfer complex at MAMs—were identified, and their interaction with FLVCR1a was independently confirmed by proximity ligation assay (PLA) and Co-Immunoprecipitation (Co-IP).

Given the essential role of the IP3R3-VDAC complex in regulating calcium flux between the ER and mitochondria, we examined whether FLVCR1a deficiency could impact MAMs function. Strikingly, loss of FLVCR1a led to a marked reduction in ER-mitochondria contact sites and a significant impairment of calcium transfer from the ER to mitochondria in HeLa cells. Importantly, the same defects were observed in disease-relevant models, including fibroblasts from PCARP/HSAN patients and *Flvcr1a-*null murine neuroprogenitors.^14,31^ Together, these findings not only reveal a novel intracellular role for FLVCR1a but also strongly suggest that its dysfunction at MAMs may directly contribute to the pathogenesis of FLVCR1-related disorders.

These findings also raise the intriguing possibility that FLVCR1a’s function at MAMs may be mechanistically linked, directly or indirectly, to its role in choline and ethanolamine transport. Further studies will be necessary to determine whether these two functions are independent or if their interplay contributes synergistically to the pathogenesis of FLVCR1-related disorders.

#### FLVCR1a role in mitochondrial metabolism

Accumulating evidence implicates FLVCR1a as an important regulator of mitochondrial metabolism across multiple pathophysiological settings. This concept is mechanistically consistent with FLVCR1a functions in heme homeostasis, choline/ethanolamine transport and Ca^2+^ handling, three processes that converge on mitochondrial integrity and bioenergetics.^31^ Multi-omics and pathway-level analyses support a mitochondrial stress signature in FLVCR1 deficiency. In particular, a GSEA analysis predicted a phenotype of ISR activation through the mitochondrial stress response pathway.^50^ Cell-based metabolomic, proteomic and lipidomic analysis suggested mitochondrial dysfunction mainly characterized by mitochondrial membrane depolarization, ultrastructural and cristae disorganization of mitochondria. Furthermore, metabolomics performed on E11.5 Flvcr1-null embryos revealed a significant reduction in choline, betaine and phosphocholine levels together with mitochondrial dysfunction markers, ISR activation, decreased abundance of mitochondrial proteins and perturbed mitochondrial cristae density and ultrastructure.^50^ Interestingly, impaired energetic metabolism, with reduced TCA cycle enzyme activity, reduced mitochondrial ATP levels and increased lipid peroxidation levels was observed in Flvcr1a-null neuronal progenitors,^14^ in *flvcr1a* morphants^31^ and in patient-derived fibroblasts.^14,31^ Furthermore, alteration of mitochondrial morphology was documented in both retina- and rods-specific *Flvcr1*-knockout mice.^40^ Collectively, these observations position mitochondrial dysfunction as a central component of FLVCR1-related disease pathogenesis. However, it remains unclear how FLVCR1a’s distinct functions in the regulation of heme metabolism, choline/ethanolamine uptake and Ca^2+^ homeostasis are integrated and whether they contribute independently or interdependently to the metabolic phenotype. Preliminary work in patient-derived fibroblasts begins to clarify these links. Of note, patient-derived fibroblasts are characterized by decreased ALAS1 activity, reduced choline levels and altered membrane fluidity, impaired MAMs’ structure and function, and overt mitochondrial failure.^31^ Importantly, short-term choline supplementation fully rescued membrane fluidity but exerted only a modest effect on energetic metabolism.^31^ Conversely, stimulation of heme synthesis using ALA to bypass ALAS1 inhibition improved mitochondrial bioenergetics.^31^ Finally, overexpression of the mitochondrial calcium uniporter (MCU) located on the inner mitochondrial membrane fully restored bioenergetic failure.^14,31^ Together, these findings establish a functional connection between FLVCR1a-dependent pathways and mitochondrial performance, while also highlighting the need for future studies to determine whether these pathways are mechanistically coupled (i.e. part of a single axis) or represent parallel, partially compensatory inputs into mitochondrial homeostasis.

#### FLVCR1b function

The second isoform encoded by the *FLVCR1* gene, FLVCR1b, is still poorly characterized both at structural and functional levels. The cryo-EM structure of FLVCR1b has not been resolved yet. As FLVCR1b contains just six hydrophobic transmembrane domains, we previously hypothesized that FLVCR1b may homodimerize, potentially forming a transporter with 12 transmembrane domains, analogous to FLVCR1a. This hypothesis is in line with observations from other MFS proteins that possess six transmembrane domains and can form functional homodimers.^33^ However, it is important to note that this remains a theoretical model, as direct experimental evidence supporting FLVCR1b dimerization is currently lacking. Regarding the substrate specificity, we originally proposed FLVCR1b as a mitochondrial heme exporter^33^ and a recent study supported its role in the delivering heme from mitochondria to the heme chaperone GAPDH.^54,55^ However, studies directly investigating the substrate specificity of FLVCR1b are still lacking. The structural similarity between FLVCR1a and FLVCR1b, together with the hypothesis of a potential homodimerization of FLVCR1b, inevitably poses the question of whether FLVCR1b might be also involved in the transport of choline and/or in the regulation of MAMs structure and function. As pathogenetic variants can also affect FLVCR1b, detailed investigations of FLVCR1b function are mandatory to improve our understanding of disease pathogenesis.

## Discussion

Over the past decade, numerous studies have demonstrated that mutations in the *FLVCR1* gene are responsible for a spectrum of autosomal recessive disorders, predominantly impacting the nervous system. The affected individuals might experience the dysfunction of three specific sensory modalities (vision, proprioception and nociception) or severe developmental defects, often incompatible with life, including microcephaly and congenital hydrocephalus. Given the increasing number of patients identified with FLVCR1-related diseases, the *FLVCR1* gene should be included as a standard component of neuropathy, retinopathy and neurodevelopmental diseases gene panels. Different variants associated with mild (Tables 1–4) or severe (Table 5) clinical manifestations have been reported to date. However, despite the growing number of identified cases, establishing a clear genotype–phenotype correlation remains challenging. It is plausible that the severity of symptoms correlates with varying degrees of FLVCR1 dysfunction, but also environmental and genetic modifiers might represent important contributing factors.

Available data supports a unifying concept that FLVCR1-related disorders are primarily diseases of neuronal metabolic vulnerability. Indeed, the high energetic demand and reliance on membrane integrity in the central and peripheral nervous system may explain the peculiar sensitivity of the nervous system to FLVCR1 dysfunction. Future research should focus on elucidating the pathogenetic mechanisms underlying FLVCR1-related diseases. Interestingly, recent studies redefined FLVCR1a as a choline and ethanolamine transporter implicated in numerous biological processes, including the regulation of heme metabolism, mitochondria-ER contacts, mitochondrial Ca^2+^ handling and energetic metabolism. As both the central and peripheral nervous system strongly rely on proper regulation of lipid and energetic metabolism, it is not surprising that *FLVCR1* mutations predominantly affect the nervous system. However, how the diverse functions of FLVCR1a are integrated and contribute to disease pathogenesis remains poorly understood. This knowledge gap largely stems from the lack of studies on cellular and animal models that faithfully reproduce the clinical features of the disease. To date, mechanistic insights derived from studies on the overexpressed mutant forms of the transporter and patients’ fibroblasts, but advanced studies on cellular and animal models that faithfully reflect tissue-specific pathology are not yet available. In recent years, the repertoire of animal models recapitulating selected disease features has expanded, improving opportunities to dissect FLVCR1 biology *in vivo*. However, a mouse model that mimics FLVCR1-related sensory neuropathy is still needed. Overall, these models will be essential to refine our understanding of disease mechanisms and to enable preclinical evaluation of therapeutic strategies. For instance, choline supplementation has been proposed as a safe therapeutic approach but the strength of the current evidence supporting its efficacy remains limited. The rationale is mainly derived from *in vivo* observations reporting a slight delay in embryonic lethality in Flvcr1-null embryos and minor improvements in retinal morphology in rod-specific Flvcr1 knockout mice^40^, without clear demonstration of sustained functional rescue. Therefore, the translational relevance of choline supplementation and its therapeutic benefit *in vivo* remain uncertain and should be tested systematically in appropriate disease models.

Importantly, additional studies addressing the possible interplay between FLVCR1a’s diverse functions particularly its role in phospholipid metabolism, MAMs integrity, and calcium regulation may open new horizons for investigation, ultimately providing a more integrated view of disease pathogenesis and guiding the design of innovative therapeutic approaches. We envision that the integration of OMICs technologies, including transcriptomics, metabolomics, and lipidomics, in these new experimental models of the disease, will provide a comprehensive view of the molecular pathways affected by FLVCR1 dysfunction and help identify novel targets for intervention.

## Data Availability

Data sharing is not applicable to this article as no new data were created or analysed.
